# What are the reasons for the repeated failures of clinical trials with anti-amyloid drugs for AD treatment?

**DOI:** 10.1590/1980-5764-DN-2025-E001

**Published:** 2025-02-17

**Authors:** Orestes Vicente Forlenza, Breno José Alencar Pires Barbosa

**Affiliations:** 1 Universidade de São Paulo, Faculdade de Medicina, Hospital das Clínicas, Instituto de Psiquiatria, Laboratório de Neurociências, São Paulo SP, Brazil.; 2 Universidade Federal de Pernambuco, Centro de Ciências Médicas, Área Acadêmica de Neuropsiquiatria, Recife PE, Brazil.

The recent position article from the Scientific Department of Cognitive Neurology and Aging of the Brazilian Academy of Neurology not only provides recommendations for the appropriate use of anti-amyloid therapies in Brazil but also aims to raise critical points that limit potential approval for clinical use in the country^1^. Among the main topics of discussion is the need to explore the reasons for the repeated failures of anti-amyloid therapies.

A swift analysis of the available trials involving anti-amyloid immune compounds prompts that the clinical benefits have fallen short of expectations, despite accomplishing the expected biological outcome, *i.e*., amyloid clearance from the brain. Methodological shortcomings have been proposed to explain the limited or lacking efficacy observed in several well-conducted, large-scale randomized controlled trials (RCTs) using these compounds. Criticism ranges from identifying flaws in experimental design to questioning core neurobiological assumptions within the disease model^2^. In the bottom line, some authors suggest that the “amyloid cascade hypothesis” may be incorrect or insufficient to fully explain the pathogenesis of sporadic Alzheimer Disease (AD). Addressing these concerns is essential for improving the success rate in AD drug development, particularly in passive immunotherapy trials, which remain the most promissing disease-modifying approach to date^3,4^.

## Hypothesis 1: flawed experimental design

At least two critical concerns regarding the possibility of experimental flaws have been raised. First, anti-amyloid treatments must be administered during the early stages of the disease process, *i.e*., before the establishment of severe neurodegeneration secondary to AD pathology, which refers to downstream pathological events that pertain to the amyloid cascade. The timing of treatment implementation along the disease continuum is a crucial determinant of success. Results from failed phase 3 trials indicate that the interventions are ineffective in patients with AD who already present symptoms of full-blown dementia, even at mild to moderate stages^5,6,7^. In other words, at the dementia stage of AD, it may be too late (in the disease continuum) for amyloid removal to yield any clinical improvement. More promising, though not definitive, results have been reported by more recent trials designed to address disease modification among patients with incipient AD^8,9,10^. These trials enrolled cases of mild cognitive impairment (MCI) due to AD, or even at earlier stages of asymptomatic AD, with diagnostic confirmation of prodromal or preclinical disease according to validated AD biomarkers. Delivering disease-modifying interventions during pre-dementia stages represents a pragmatic shift in the management of AD, *i.e*., from treatment to prevention or attenuation of dementia.

Despite this shift, predicting treatment responses in patients with incipient disease remains complex. Recent evidence from *post-hoc* analyses of studies using compounds such as donanemab, lecanemab, and gantenerumab — presented at the 16^th^ Clinical Trials on Alzheimer’s Disease Conference (CTAD), held in Boston, USA, October 24-27, 2023 — suggests that clinical (cognitive) benefits are more significant when treatment is delivered at the earliest stages of the pathological process, *i.e*., at some point between amyloid deposition and the onset of neurofibrillary degeneration. Responders are typically individuals with asymptomatic or oligosymptomatic AD with proven intracerebral amyloid burden but limited tangle pathology, as shown by lower tau-PET or plasma phospho-tau levels at baseline (www.alzforum.org, “Treat Before ‘Aβ Bothers Tau,’ Scientists Say at CTAD”; accessed on Nov 15^th^, 2023). On the other hand, the failure of gantenerumab and solanezumab trials to prevent cognitive decline in asymptomatic individuals with dominantly inherited AD highlights significant challenges, emphasizing the complexity of this approach^11^. Furthermore, including asymptomatic subjects in immunotherapy trials raises ethical concerns, considering that most asymptomatic subjects with isolated amyloidosis will not clinically manifest the symptoms of the disease during the follow-up period^12^.

Another methodological limitation is that the duration of the intervention in most trials may have been insufficient to yield good discrimination between experimental and control groups concerning primary (clinical) outcome measures. AD is an insidious disease, with cognitive decline occuring at a slow pace, particularly in sporadic, late-onset cases. Global cognitive function in patients with very mild cognitive deficits within the MCI continuum may outlive the duration of the trial, reducing the likelhood of detecting objective differences between treatment groups by the study’s endpoint. Therefore, the dynamics of the long-term therapeutic effects of AD immunotherapy remain unclear. Detectable clinical benefits from amyloid removal from the brain require extended follow-up periods. Although possible in small, open-label studies and clinical practice, such long-term controlled studies are often unrealistic to be conducted in the experimental setting. In practical terms, it is a requirement that jeopardizes the feasibility of large-scale trials.

Nonetheless, exciting analyses are emerging from open-label extension (OLE) studies with several anti-amyloid drugs. Although the findings are not compelling, clinical deterioration rates after treatment discontinuation often mirror those observed in placebo groups. Rather than reinforcing the expected disease-modifying pattern, long-term responses to anti-amyloid immunocompounds largely resembles the curves seen with symptomatic anti-dementia drugs. For instance, an OLE study of lecanemab monitored clinical parameters after the primary study endpoint; similar between-group differences were observed through an intervening off-treatment period of 24 months, suggesting that, after discontinuation, treated patients continued to deteriorate at a similar rate as untreated ones^13^.

## Hypothesis 2: the selection of participants must be improved

Even though most clinical trials have systematically adopted biomarker information (Aβ and tau) for patient enrollment, other sources of biological heterogeneity may have endured in study samples, rendering heterogeneous treatment groups and, hence, mixing responders with non-responders. Therefore, limited or insufficient biomarker instrumentation, precluding an optimized identification of eligible patients, is another possible methodological flaw that might help explain some of these failures. As stated in the previous topic, staging tau pathology and the actual characterization of amyloid burden may also be needed to better identify responders among cases with minimal neurodegeneration. Some effort has been made in this direction. Donanemab’s trial, for example, used PET-tau positivity as an inclusion criterion and differentiated individuals based on low/medium and high tau pathology. Counterintuitively, the results showed a statistical effect regardless of tau pathology severity^10^. Additionally, the presence of intracerebral amyloid *per se* may not suffice to predict clinical response, given the numerous concurrent pathological processes downstream of amyloid and beyond tau-related neurodegeneration, such as neuroinflammation, oxidative stress, mitochondrial dysfunction, lysosomal dysfunction, insulin resistance, lipid abnormalities, and other putative mechanisms^14^.

Furthermore, the pathogenic process in AD is intertwined with multiple midlife and late-life risk factors, which interact with components of the amyloid cascade. These interactions exacerbate neurofibrillary degeneration by upregulating inflammation and oxidative stress while downregulating neuroprotective and restorative responses, causing additional neuronal and synaptic damage. This complex pathophysiological process unfolds long before the onset of cognitive decline. Therefore, conceptualizing AD as a simple consequence of amyloid overproduction and accumulation in the brain overlooks the intricate interplay between amyloid and other pathological or age-related mechanisms. While such amodel may be effective in controlled preclinical models, it may not accurately reflect real-life scenarios or clinical practice. This oversimplification poses significant challenges in accounting for all intervening factors during clinical experimentation.

## Hypothesis 3: better immunocompounds are needed

Improvements in immunotherapeutic compounds *per se* are likely necessary. The drugs tested for treating AD target distinct epitopes and conformations of amyloid-beta (*e.g*., Aβ monomers, oligomers, protofibrils, fibrils, and plaques)^15^. Certain anti-amyloid compounds have been argued to be ineffective in removing the particularly toxic forms of the peptide, highlighting the need for further development to identify the specific Aβ forms that must be targeted to yield neuroprotective effects and modify the biological trajectory of the disease^16^. It is also crucial to improve the safety profile of anti-Aβ immunotherapy, given the high incidence of amyloid-related imaging abnormalities (ARIAs) observed with most clinically tested compouds. In this regard, promising perspectives have been emerging from preclinical studies utilizing engineered anti-amyloid antibodies. Pizzo et al. have recently demonstrated that the introduction of a transferrin receptor (TfR) binding site at the tail region (Fc domain) of an anti-Aβ antibody not only improved its access to the brain and distribution within the parenchyma, but also reduced its presence in perivascular spaces, therefore minimizing the contact of the immunocompound with its target in leptomeningeal arteries. In a transgenic mouse model of AD, this modification lessened antibody binding to vascular Aβ, therefore reducing the incidence of amyloid angiopathy^17^. If replicated clinically, this effect should warrant an actual reduction of the incidence of ARIA events associated with passive anti-Aβ immunotherapy.

An important aspect has been raised in discussing why specific trials yielded positive clinical results while similarly well-conducted trials with comparable drugs produced less consistent or negative results. Secondary analyses of these trials suggest that the *rate and degree* of amyloid removal are critical determinants of success, meaning that clinical benefit appears to depend on achieving practical and sustained removal of amyloid from the brain^18^. The effectiveness of amyloid removal may help explain the clinical success of lecanemab in the CLARITY trial^9^, as well as the inconsistent results obtained with aducanumab in EMERGE and ENGAGE trials^19^, and the largely negative clinical results with gantenerumab in the GRADUATE trials^20^. These findings suggest that therapuetic benefits emerge only after intracerebral amyloid content is reduced beyond a critical level. This would implicate longer treatments, higher doses, or more effective amyloid-lowering compounds. In addition, understanding the epitope-binding properties of these compounds is essential for evaluating their potential to deliver therapeutic effects and induce adverse event^18^.

## Hypothesis 4: biological assumption is insufficient or incorrect

Much of the knowledge on disease modification in AD derives from animal models where anti-amyloid agents attenuated intracerebral amyloidosis and respective downstream effects. In these models, biological improvements were accompanied by behavioral changes that parallel cognitive benefits. Such a large and compelling body of evidence from experimental models has nonetheless created a “translational gap” between animal and human studies^21^. Therefore, high expectations based on preclinical studies have often been utterly frustrated by the actual outcomes of clinical trials. Improved animal models may be necessary to better depict the biological complexity of sporadic AD and more accurately predict the outcomes in human trials.

AD is undeniably a multifaceted disease. Hence, considering alternative (non-amyloid) disease pathways may uncover new molecular targets which may be more amenable to pharmacological manipulation. The neurobiological complexity of AD will most likely support the development of combined approaches and multi-target compounds^22^. Although amyloid plaques, along with neurofibrillary tangles, represent pathological hallmarks of AD, it remains unclear whether the actual removal of amyloid from the brain will decisively modify the disease’s natural progression. According to the amyloid hypothesis of AD, abnormal Aβ overproduction and accumulation are prime molecular events that trigger downstream neurodegenerative effects, ultimately leading to cognitive decline. On the other hand, evidence suggests that amyloid production may represent a protective response to neurotoxic insults, such as inflammatory and immune-mediated processes. This hypothesis posits that Aβ secretion by neural cells may occur as a compensatory mechanism in response to primary insults, challenging the amyloid cascade hypothesis. This notion also subsumes that soluble Aβ species would be more neurotoxic than insoluble ones (*e.g*., oligomeric and protofibrillary Aβ, respectively), and their removal would be more beneficial than removing the amyloid content deposited in plaques. All in all, there is still much to learn about this, given that results from clinical trials using immunocompounds that predominantly target soluble Aβ have not consistently supported this notion^23^.

In conclusion, developing effective new treatments for AD may require multiple amendments to the current state of the art. Efforts should include optimizing the recruitment processes to ensure a more accurate and specific selection of eligible patients with clinical-biological profiles that are more likely to respond to anti-amyloid drugs. Additionally, a better comprehension of treatment dynamics is needed to refine clinical trial designs, encompassing more realistic endpoints^24^. In the forthcoming years, the AD drug development pipeline will probably incorporate a diversification of molecular targets, combined therapies, and non-pharmacological treatments to simultaneously intervene in multiple disease mechanisms of the disease. The timing of intervention will be crucial to avoid recapitulating the failure of recent trials with anti-amyloid compounds. The complexity of the disease, including, but not limited to, amyloid accumulation, highlights the necessity of multi-target and multi-modal interventions, reducing the gap between experimental and clinically-driven interventions with disease-modifying strategies. 

## References

[1] Barbosa BJAP, Resende EPF, Castilhos RM, Borelli WV, Frota NAF, Balthazar MLF, Amato ACS (2024). Use of anti-amyloid therapies for Alzheimer’s disease in Brazil: a position paper from the Scientific Department of Cognitive Neurology and Aging of the Brazilian Academy of Neurology.. Dement Neuropsychol.

[2] Loureiro JC, Pais MV, Stella F, Radanovic M, Teixeira AL, Forlenza OV (2020). Passive antiamyloid immunotherapy for Alzheimer’s disease. Curr Opin Psychiatry.

[3] Cummings JL, Morstorf T, Zhong K (2014). Alzheimer’s disease drug-development pipeline: few candidates, frequent failures. Alzheimers Res Ther.

[4] Kim CK, Lee YR, Ong L, Gold M, Kalali A, Sarkar J (2022). Alzheimer’s Disease: key insights from two decades of clinical trial failures. J Alzheimers Dis.

[5] Salloway S, Sperling R, Fox NC, Blennow K, Klunk W, Raskind M (2014). Two phase 3 trials of bapineuzumab in mild-to-moderate Alzheimer’s disease. N Engl J Med.

[6] Doody RS, Thomas RG, Farlow M, Iwatsubo T, Vellas B, Joffe S (2014). Phase 3 trials of solanezumab for mild-to-moderate Alzheimer’s disease. N Engl J Med.

[7] Honig LS, Vellas B, Woodward M, Boada M, Bullock R, Borrie M (2018). Trial of solanezumab for mild dementia due to Alzheimer’s disease. N Engl J Med.

[8] Mintun MA, Lo AC, Wessels AM, Ardayfio PA, Andersen SW (2021). Donanemab in early Alzheimer’s disease. N Engl J Med.

[9] van Dyck CH, Swanson CJ, Aisen P, Bateman RJ, Chen C, Gee M (2023). Lecanemab in early Alzheimer’s disease. N Engl J Med.

[10] Sims JR, Zimmer JA, Evans CD, Lu M, Ardayfio P, Sparks JD (2023). Donanemab in early symptomatic Alzheimer disease: the TRAILBLAZER-ALZ 2 randomized clinical trial. JAMA.

[11] Salloway S, Farlow M, McDade E, Clifford DB, Wang G, Llibre-Guerra JJ (2021). A trial of gantenerumab or solanezumab in dominantly inherited Alzheimer’s disease. Nat Med.

[12] Brookmeyer R, Abdalla N (2018). Estimation of lifetime risks of Alzheimer’s disease dementia using biomarkers for preclinical disease. Alzheimers Dement.

[13] McDade E, Cummings JL, Dhadda S, Swanson CJ, Reyderman L, Kanekiyo M (2022). Lecanemab in patients with early Alzheimer’s disease: detailed results on biomarker, cognitive, and clinical effects from the randomized and open-label extension of the phase 2 proof-of-concept study. Alzheimers Res Ther.

[14] Cummings J, Zhou Y, Lee G, Zhong K, Fonseca J, Cheng F (2022). Alzheimer’s disease drug development pipeline: 2022.. Alzheimers Dement (N Y).

[15] van Dyck CH (2018). Anti-amyloid-β monoclonal antibodies for Alzheimer’s disease: pitfalls and promise.. Biol Psychiatry.

[16] Söderberg L, Johannesson M, Nygren P, Laudon H, Eriksson F, Osswald G (2023). Lecanemab, Aducanumab, and Gantenerumab – binding profiles to different forms of amyloid-beta might explain efficacy and side effects in clinical trials for Alzheimer’s disease. Neurotherapeutics.

[17] Pizzo ME, Plowey ED, Khoury N, Kwan W, Abettan J, DeVos SL Engineering anti-amyloid antibodies with transferrin receptor targeting improves brain biodistribution and mitigates ARIA. bioRxiv.

[18] Hardy J, Mummery C (2023). An anti-amyloid therapy works for Alzheimer’s disease: why has it taken so long and what is next?. Brain.

[19] Schneider LS (2022). Editorial: Aducanumab trials EMERGE but don’t ENGAGE.. J Prev Alzheimers Dis.

[20] Bateman RJ, Smith J, Donohue MC, Delmar P, Abbas R, Salloway S (2023). Two phase 3 trials of gantenerumab in early Alzheimer’s disease. N Engl J Med.

[21] Gregory S, Saunders S, Ritchie CW (2022). Science disconnected: the translational gap between basic science, clinical trials, and patient care in Alzheimer’s disease. Lancet Healthy Longev.

[22] van Bokhoven P, Wilde A, Vermunt L, Leferink PS, Heetveld S, Cummings J (2021). The Alzheimer’s disease drug development landscape. Alzheimers Res Ther.

[23] Karran E, De Strooper B (2022). The amyloid hypothesis in Alzheimer disease: new insights from new therapeutics. Nat Rev Drug Discov.

[24] Schneider LS (2023). What the gantenerumab trials teach us about Alzheimer’s treatment. N Engl J Med.

